# Analysis of rs1864182 and rs1864183 variants in *ATG10* gene and antineutrophil cytoplasmic autoantibody‐associated vasculitis in Chinese Guangxi population

**DOI:** 10.1002/jcla.24193

**Published:** 2021-12-27

**Authors:** Shanshan Huang, Jinlan Rao, Jingsi Wei, Qunshen Huang, Yan Zhu, Wei Li, Chao Xue

**Affiliations:** ^1^ The Second Clinical Medical College of Guangxi Medical University Nanning China; ^2^ The First Affiliated Hospital of University of South China Hengyang China; ^3^ Department of Nephrology The Second Affiliated Hospital of Guangxi Medical University Nanning China

**Keywords:** antineutrophil cytoplasmic antibody‐associated vasculitis, autophagy‐related protein 10, GMDR, interaction, polymorphism

## Abstract

**Objectives:**

To investigate the association of autophagy‐associated gene 10 (*ATG10*) gene polymorphisms (rs1864182 and rs1864183) with antineutrophil cytoplasmic autoantibody (ANCA)‐associated vasculitis (AAV) in Chinese Guangxi population.

**Methods:**

The single nucleotide polymorphisms (SNPs) of *ATG10* rs1864182 and rs1864183 in 395 participants (195 AAVs and 200 healthy controls) were genotyped. Generalized multiple dimensionality reduction (GMDR) was used to analyze the SNP‐SNP interactions among two SNPs of *ATG10* gene and other SNPs of autophagy gene previously studied by our research team.

**Results:**

In this study, we found that the two *ATG10* SNPs were not associated with AAV risk in Chinese Guangxi population. However, there were statistically significant differences in the incidence of hemoptysis, hematuria, and proteinuria among the three genotypes of *ATG10* rs1864182 and rs1864183 (*p* < 0.05). Moreover, permutation test of GMDR suggested that immunity‐related GTPase M(*IRGM*) rs4958847, autophagy‐associated gene 7 (*ATG7*) rs6442260, *ATG7* rs2594966, *ATG10* rs1864183, protein kinase B(*AKT2*) rs3730051, and *AKT2* rs11552192 might interact with each other in the process of developing AAV (*p* < 0.05).

**Conclusions:**

Our results indicated that there existed no association between *ATG10* SNPs and AAV, and SNP‐SNP interactions among *IRGM* rs4958847, *ATG7* rs6442260, *ATG7* rs2594966, *ATG10* rs1864183, *AKT2* rs3730051, and *AKT2* rs11552192 may confer AAV risk in the Chinese Guangxi population.

## Graphical abstract

Although we do not report the association between ATG10 rs1864182 and rs1864183 gene polymorphisms and AAV in our population, SNP‐SNP interactions in autophagy genes family may increase the susceptibility risk of individuals to AAV.

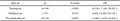

## 1 INTRODUCTION

Antineutrophil cytoplasmic autoantibody (ANCAs)‐associated vasculitis (AAV) is a sort of autoimmune disease which affects mainly arterioles, capillaries, and systemic primary blood vessels,^1^ involving eosinophilic granulomatosis with polyangiitis (EGPA), microscopic polyangiitis (MPA), and granulomatosis with polyangiitis (GPA).^2^ The precise etiology of AAV remains unclear, but infection, autoimmune diathesis, and genetic are considered as the crucial triggers for AAV currently.^3^, ^4^ In some large genome‐wide association studies (GWAS), major histocompatibility complex (*HLA*), protein tyrosine phosphatase non‐receptor type 22 (*PTPN22*), cytotoxic T‐lymphocyte‐associated protein 4 (*CTLA*‐*4*), interleukin 10 (*IL*‐*10*), and Toll‐like receptor 9 (*TLR9*) have been found to be related to AAV.^5^, ^6^In a recent GWAS of EGPA, MPO‐ANCA(+) subset is closely related to major histocompatibility complex, class II, DQ(*HLA*‐*DQ*), while MPO‐ANCA(−) subgroup is related to non‐*HLA* regions.^7^In addition, different clinical subtypes of AAV have different genetic backgrounds. GPA is associated with major histocompatibility complex, class II, DP 1 (*HLA*‐*DP1*), EGPA with major histocompatibility complex, class II, and DR beta 4 (*HLA*‐*DRB4*), while MPA is closely related to *HLA*‐*DQ*.^8^

Autophagy is a complex cellular mechanism, which mainly maintains the homeostasis and integrity of cells and tissues through misfolded proteins and the degradation of infectious factors. An increasing number of studies^9^, ^10^, ^11^ have shown that autophagy is involved in various immune processes, including the clearance of intracellular bacteria, the presentation of autoantigen and the production of cytokines, and the survival of lymphocytes, which indicates that autophagy plays an obvious and important role in the pre‐adaptive and adaptive immune responses. Moreover, genome‐wide association studies (GWAS) identified that autophagy‐related gene polymorphisms have been implicated in the pathogenesis of a variety of autoimmune and inflammatory diseases, such as systemic lupus erythematosus (SLE), rheumatoid arthritis (RA), inflammatory bowel disease (IBD), and multiple sclerosis (MS).^12^, ^13^, ^14^ However, the role of autophagy‐associated gene 10 (*ATG10*) gene polymorphism in AAV still lacks related research. In our study, two single nucleotide polymorphisms (SNPs) of *ATG10* gene were selected to preliminarily explore the relationship between gene polymorphism and AAV patients in Guangxi population. Besides, we used generalized multiple dimensionality reduction (GMDR) to analyze the interaction between autophagy genes and the pathogenesis of AAV, so as to provide new ideas for the prevention and treatment of AAV.

## 2 MATERIALS AND METHODS

### 2.1 Patients and controls

In this study, a total of 195 AAV patients hospitalized in the Second Affiliated Hospital of Guangxi Medical University (formerly Western Hospital of the First Affiliated Hospital of Guangxi Medical University) from 2008 to 2021 were collected. The criteria were as follows: (1) All cases were strictly based on the criteria of the 2012 Chapel Hill International Conference on Vasculitis Nomenclature^15^; (2) patients have been diagnosed for the first time and have not yet started glucocorticoids or immunosuppressive therapy; (3) all patients are local people who have lived in Guangxi for three generations. Patients with secondary infections, SLE, RA, or any secondary vasculitis were removed. The control group was all local healthy volunteers from the same hospital in Guangxi during the same period, who were matched to the AAV group by sex, age, and ethnic background. Participants with a personal or family history of type 2 diabetes mellitus (T2DM), high blood pressure (HBP), or other chronic diseases were excluded. This study was approved by the Ethics Committee of the Second Affiliated Hospital of Guangxi Medical University (No. 2018 KY‐0100), and all subjects provided informed consent before blood collection.

### 2.2 DNA extraction

Peripheral venous blood (5 ml) from all subjects was collected by EDTA anticoagulant blood sampling vessel. Total DNA was extracted by DNA extraction kit provided by Tiangen Biochemical Technology. All operations were conducted in strict accordance with the instructions. DNA samples with absorbance value (A260/280 nm) ranging from 1.5 to 2.0 at a concentration >50 ng/μl were stored in a refrigerator at −80℃ for subsequent experiments.

### 2.3 SNP selection

The locus information of *ATG10* gene was downloaded from 1000 genomes (http://grch37.ensemble.org/), and the SNP was screened by Haploview 4.2 software. The selection criteria were as follows: (A) minimum allele frequency (MAF) ≥ 0.05 and (B) Hardy‐Weinberg equilibrium (HWE) test *p* > 0.05. In addition, we used the National Center for Bioinformatics (NCBI, https://www.ncbi.nlm.nih.gov/snp/) to investigate the location of SNP, MAF, and other related information.

### 2.4 SNP genotyping

In this study, multiplex PCR combined with high‐throughput sequencing technology (Sangon Biotech) was used to detect the genotype of SNPs. Briefly, we design and synthesize a primer pool (containing two SNP loci of *ATG10* gene), and then, we use a two‐step PCR to amplify the target SNPs sequence and prepare a compatible Illumina sequencing library. The first round of PCR system included: DNA template (10 ng/μl, 2 μl); upstream primer pool (10 μmol/L, 1 μl); downstream primer pool (10 μmol/L, 1 μl); and 2×PCR Ready Mix 15 μl (total volume 25 μl; Kapa HiFi Ready Mix). The reaction steps were performed on the PCR instrument (Bio‐Rad, T100TM) using the prepared reaction system. PCR product size was detected using 1% agarose gel electrophoresis, and AMPure XP magnetic beads were used to purify and recycle PCR products. The method of obtaining a library with molecular tags was to perform a second PCR using the first PCR product as a template. The reaction system was performed according to the following procedure: The first PCR product was used as template (10 ng/μl, 2 μl), universal P7 primer (including molecular label, 10 μmol/L, 1 μl); universal P5 primer (10 μmol/L, 1 μl), and PCR Ready Mix 15 μl (total volume 30 μl). A new round of PCR was conducted using the prepared reaction system. AMPure XP magnetic beads are used to purify and recycle the final product. The products were mixed in equal quantities and sequenced by using a HiSeq Xten sequencer (Illumina).

### 2.5 GMDR analysis

We evaluated the interactions among susceptible SNPs of autophagy gene family by using GMDR 0.7 software. The test level α = 0.05, and *p* < 0.05 was considered statistically significant.

### 2.6 Statistical analysis

Chi‐squared test was used to estimate the deviations from Hardy‐Weinberg equilibrium (HWE) of selected *ATG10* SNPs. SPSS 23.0 statistical software (IBM) was performed for statistical analysis, and *p* < 0.05 was considered statistically significant. The enumeration data are expressed in percentage, and the measurement data are expressed as mean (SD). Odds ratios (ORs) and 95% confidence intervals (CIs) were carried out. Linkage disequilibrium (LD) test and haplotype analysis were conducted by SHEsis online software (http://analysis.bio‐x.cn/myAnalysis.php; Shi and He 2005).^16^

## 3 RESULTS

### 3.1 Demographic characteristics and comparison of participants

In this study, a total of 395 subjects were involved, of which 195 were AAV cases and 200 were healthy controls. There was no statistically significant difference in the average age and gender composition between the two groups (*p* > 0.05), and they were comparable. The demographic characteristics of each participant are summarized in Table 1.

**TABLE 1:** General data of participants

Characteristic	AAVs	Controls	*Χ* ^2^/t	*p*
Total number	195	200	‐	‐
Male/Female	71/124	78/122	0.282	0.595
Age (years)	54.59 ± 14.96 (19–82)	52.14 ± 11.60 (18–81)	1.825	0.069
SBP (mmHg)	135.7 ± 21.5	123.0 ± 10.5	7.493	0.000
DBP (mmHg)	78.5 ± 12.7	74.9 ± 8.4	3.292	0.001
SCR (μmol/L)	386.378 ± 384.171	68.190 ± 14.783	11.706	0.000
24 h UPR (g/L)	1584.917 ± 1769.287	20.707 ± 21.234	131.661	0.000

Note

This data type is measurement data. *p*‐value: the comparisons were made between AAV patients and controls using Student's *t* test or chi‐square (*χ* ^2^) test. *p* < 0.05 was considered statistically significant.

Abbreviations: 24 h UPR, 24 h of urinary protein quantitation; AAV, antineutrophil cytoplasmic autoantibody‐associated vasculitis; DBP, diastolic blood pressure; SBP, systolic blood pressure; SCR, serum creatinine.

### 3.2 Allele frequency, genotype distribution, and HWE analysis

**FIGURE 1:**
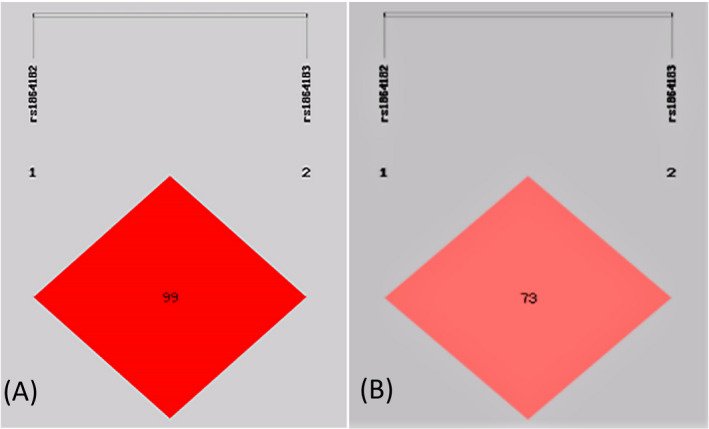
D′ and *R* ^2^ value heat map of linkage disequilibrium in SNPs of *ATG10* gene. Note: A: D′ = 0.99; B: *R* ^2^ = 0.73. Linkage disequilibrium (LD) test and haplotype analysis were carried out by SHEsis online software (http://analysis.bio‐x.cn/myAnalysis.php; Shi and He 2005). Abbreviations: SNP, single nucleotide polymorphisms; ATG10, autophagy‐associated gene 10

The genotype distributions of the two *ATG10* SNPs in controls were in HWE (all *p* > 0.05). As shown in Table 2, the alleles and genotypes did not show any significant difference between AAV patients and controls. We also demonstrated that *ATG10* rs1864182 and rs1864183 were in complete linkage disequilibrium, as shown in Figure 1 (D′ = 0.99, *R* ^2^ = 0.73).

**TABLE 2:** Association analysis for 2 target SNPs within ATG10 gene and AAV risk

SNP	Genotype frequency (%)			Allele (%)		OR (95% CI)
rs1864182	AA	AC	CC	A	C	
AAVs	162 (83.1)	31 (15.9)	2 (0.5)	355 (91.0)	35 (9.0)	1.286 (0.807–2.048)
Controls	158 (79.0)	39 (19.5)	3 (1.5)	355 (88.7)	45 (11.3)	
*χ* ^2^		1.101		1.124		
*p*		0.577		0.289		
rs1864183	CC	CT	TT	C	T	
AAVs	2 (1.0)	43 (22.1)	150 (76.9)	47 (12.1)	343 (87.9)	0.808 (0.535–1.221)
Controls	6 (3.0)	46 (23.0)	148 (74.0)	58 (14.5)	342 (85.5)	
*χ* ^2^	2.052			1.027		
*p*	0.359			0.311		

Note

This data type is enumeration data. *p*‐value: comparisons were made between AAV patients and controls using chi‐squared (*χ* ^2^) test. *p* < 0.05 was considered statistically significant.

Abbreviations: 95% CI, 95% confidence interval; AAV, antineutrophil cytoplasmic autoantibody‐associated vasculitis; OR, odds ratios.

### 3.3 Correlations between genotypes and clinical characteristics

As shown in Table 3, there were statistically significant differences in the incidence of hemoptysis, hematuria, and proteinuria among the three genotypes of *ATG10* (rs1864182 and rs1864183; *p* < 0.05). No remarkable association was observed between the two SNPs and white blood cell (WBC), neutrophil count, hemoglobin, creatinine, IgG, IgA, and IgM (*p* > 0.05), which is displayed in Table 4.

**TABLE 3:** Comparison of genotypes and clinical symptoms at two SNPs of ATG10 gene

Symptoms	rs1864182 genotype			*χ* ^2^	*p*	rs1864183 genotype			*χ* ^2^	*p*
	AA	CA	CC			TT	TC	CC		
Hematuria										
+	108 (103.4)	13 (18.1)	2 (1.5)	7.368	0.025*	102 (95.8)	19 (25.7)	2 (1.5)	9.304	0.010*
−	29 (33.6)	11 (5.9)	0 (0.5)			25 (31.2)	15 (8.3)	0 (0.5)		
Proteinuria										
+	105 (101.6)	16 (17.9)	0 (1.5)	7.175	0.028*	97 (94.1)	24 (25.4)	0 (1.5)	6.556	0.038*
−	31 (34.4)	8 (6.1)	2 (0.5)			29 (31.9)	10 (8.6)	2 (0.5)		
Hemoptysis										
+	11 (13.5)	3 (2.3)	2 (0.2)	19.486	0.000*	9 (12.5)	5 (3.3)	2 (1.8)	20.853	0.000*
−	129 (126.5)	21 (21.7)	0 (1.8)			121 (117.5)	29 (30.7)	0 (0.2)		
Edema										
+	61 (60.7)	9 (10.4)	2 (0.9)	2.950	0.229	59 (56.4)	11 (14.7)	2 (0.9)	4.506	0.105
−	79 (79.3)	15 (13.6)	0 (1.1)			71 (73.6)	23 (19.3)	0 (1.1)		

Note

This data type is enumeration data. *p < 0.05: the comparisons were made among two SNPs of ATG10 gene and clinical symptoms in AAVs using chi‐squared (*χ* ^2^) test. *p* < 0.05 was considered statistically significant.

Abbreviations: ATG10, autophagy‐associated gene 10; SNP, single nucleotide polymorphisms.

**TABLE 4:** Genotypes at two SNP of ATG10 gene AND laboratory indexes

Laboratory indexes	rs1864182 genotype			*p*	rs1864183 genotype			*p*
	AA	AC	CC		TT	TC	CC	
WBC (×10^9^/L)	8.438 ± 3.849	8.588 ± 2.972	8.600 ± 0.000	0.982	8.404 ± 3.686	8.672 ± 3.918	8.600 ± 0.000	0.932
NET (×10^12^/L)	6.850 ± 7.186	6.690 ± 2.816	5.800 ± 0.000	0.972	6.865 ± 7.331	6.679 ± 3.615	5.800 ± 0.000	0.976
HB (g/L)	81.154 ± 23.791	89.754 ± 31.463	97.000 ± 0.000	0.215	80.941 ± 23.703	88.029 ± 29.705	97.000 ± 0.000	0.245
SCR (μmol/L)	383.870 ± 379.762	422.917 ± 423.492	121.000 ± 0.000	0.558	390.047 ± 387.195	388.176 ± 384.320	121.000 ± 0.000	0.691
IgG (g/L)	14.584 ± 6.048	13.550 ± 3.988	18.440 ± 0.000	0.455	14.640 ± 6.106	13.611 ± 4.317	18.440 ± 0.000	0.421
IgA (g/L)	2.526 ± 1.430	2.429 ± 1.020	1.470 ± 0.000	0.540	2.508 ± 1.465	2.522 ± 0.968	1.470 ± 0.000	0.567
IgM (g/L)	1.288 ± 1.591	1.121 ± 0.650	0.450 ± 0.000	0.654	1.156 ± 0.711	1.634 ± 2.862	0.450 ± 0.000	0.199

Note

This data type is measurement data. *p*‐value: the comparisons were made among two SNPs of ATG10 gene and laboratory indexes in AAVs by using Kruskal‐Wallis test. *p* < 0.05 was considered statistically significant.

Abbreviations: ATG10, autophagy‐associated gene 10; HB, hemoglobin; IgA, immunoglobulin a; IgG, immunoglobulin g; IgM, immunoglobulin m; NEUT, neutrophil count; SCR, serum creatinine; SNP, single nucleotide polymorphisms; WBC, white blood cell.

### 3.4 GMDR analysis

We detected a significant six‐locus model including *ATG10* rs1864183, immunity‐related GTPase M(*IRGM*) rs4958847, autophagy‐associated gene 7 (*ATG7*) rs6442260, *ATG7* rs2594966, protein kinase B(*AKT2*) rs3730051, and *AKT2* rs11552192 (*p* = 0.011), suggesting a potential SNP‐SNP interaction among *ATG10* rs1864183, *IRGM* rs4958847, *ATG7* rs6442260, *ATG7* rs2594966, *AKT2* rs3730051, and *AKT2* rs11552192. The cross‐validation consistency of six‐locus model was 9/10, and the testing accuracy was 0.7885 (1000 permutation tests *p* > 0.05; Table 5).

**TABLE 5:** GMDR analysis for the best interaction combination model

Data set	*χ* ^2^	*p*	OR (95% CI)
Training set	64.196	0.000*	14.336 (7.103~28.932)
Validation set	1.515	0.218	4.820 (0.585~39.710)
The whole data set	69.134	0.000*	13.480 (6.978~26.043)

Note

The best interaction combination model: ATG10 rs1864183, IRGM rs4958847, ATG7 rs6442260, ATG7 rs2594966, AKT2 rs3730051, and AKT2 rs11552192. *p < 0.001: we evaluated the interactions among susceptible SNPs of autophagy gene family by using GMDR 0.7 software. *p* < 0.05 was considered statistically significant.

Abbreviations: 95% CI, 95% confidence interval; GMDR, generalized multiple dimensionality reduction; OR, odds ratios.

## 4 DISCUSSION

Autophagy plays a key role in the activation of innate and adaptive immune responses, the elimination of dead cells, the presentation of autoantigens, and the regulation of lymphocyte development, survival, and proliferation. *ATG10* encodes autophagy E2 enzyme and interacts with *ATG7* to recruit ubiquitin‐like molecule (autophagy‐associated gene 12, *ATG12*) and participate in the conjugation reaction of *ATG12*‐*ATG5* (autophagy‐associated gene 5).^17^ Minmingzheng et al. reported that *ATG10* rs4703863 was associated with the susceptibility of autoimmune disease Vogt‐Koyanagi‐Harada (VKH) syndrome.^18^ In recent years, a study conducted by Sha et al. suggested that autophagy is involved in the formation of neutrophils extracellular trap net which is an important link in the pathogenesis of AAV.^19^ It was found that the concentration of interleukin 8 (IL‐8) in neutrophil supernatant induced by ANCA further activation of macrophage migration inhibitory factor (MIF) was significantly increased.^20^ Circulating IL‐8 stimulates the production of ANCA‐activated neutrophils and endothelial cells. Recent studies indicated that IL‐8 was significantly correlated with gene polymorphism of *ATG10*.^21^ Therefore, we speculated that *ATG10* gene polymorphism may be related to the susceptibility to AAV. The results of this study indicated that the collected samples were representative of the population, suggesting that *ATG10* rs1864182 and rs1864183 had polymorphisms in Guangxi population. However, there was no significant difference in allele frequency and genotype frequency between the two SNPs, suggesting that these two SNPs may not be associated with genetic susceptibility of AAV in Guangxi population.

Hematuria and proteinuria are part of the main manifestations of AAV patients. The analysis of clinical symptoms in this study found that *ATG10* rs1864182 and rs1864183 genotypes have statistically significant differences in the distribution of hematuria and proteinuria in AAV patients. As mentioned above, the genetic polymorphism of *ATG10* is related to IL‐8.^21^ P Cockwell et al. reported that the expression of IL‐8 in the glomeruli can be observed in the segment, crescent, and parietal epithelial parts of renal tissue in patients with AAV. The internal production of IL‐8 may hinder the migration of neutrophils, promote intravascular stasis, and cause glomerular endothelial cell damage.^22^ Therefore, we speculate that *ATG10* gene may affect the expression of IL‐8 and cause kidney damage, following hematuria, proteinuria, and other clinical manifestations. Our research results also showed that the distribution of *ATG10* rs1864182 and rs1864183 genotypes in the subgroups of hemoptysis was statistically significant difference. The incidence of hemoptysis in rs1864182 AA genotype and rs1864183 TT genotype was higher than other genotypes (AC, CC of rs1864182 and TC, CC of rs1864183), revealing that rs1864182 AA genotype and rs1864183 TT genotype may be more likely to cause pulmonary vascular injury, and the A and T alleles may be risk genes. The results still need to be confirmed by more large‐scale genome‐wide studies.

GMDR is a nonparametric analysis method without specifying genetic model and interaction model.^23^ It is a powerful tool for studying multi‐gene diseases. *ATG10* single gene SNP has nothing to do with the pathogenesis of AAV. The role of a single gene is not yet fully explained the pathogenic mechanism of diseases, especially autoimmune diseases, probably because the etiology and pathogenesis are complex, its incidence is associated with the interaction of multiple genes, and thus, inheritance patterns of complex diseases could be gene‐gene interactions. The etiology and pathogenesis of AAV are complex, involving multiple genes and multiple factors, which may be caused by interaction. Therefore, the association of SNPs of *ATG10* gene and AAV cannot be carried out only by the unit point analysis of genotype and allele. It is necessary to combine the gene or the interaction between gene and environment for analysis in order to reveal the real correlation between *ATG10* and AAV accurately and comprehensively. Therefore, we used GMDR software to analyze the relationship between the interaction of two *ATG10* gene SNPs and other autophagy‐related gene SNPs studied by our research team, and the incidence of AAV. The results showed that *IRGM* rs4958847, *ATG7* rs6442260, *ATG7* rs2594966, *ATG10* rs1864183, *AKT2* rs3730051, and *AKT2* rs11552192 have interactions, suggesting that mutations in *ATG10*, *IRGM*, *ATG7*, and *AKT2* genes may interact and increase the risk of individual susceptibility to AAV. As one of the core processes of autophagy, the formation of autophagosomes is similar to ubiquitination. *ATG10* is an autophagic E2 enzyme that interacts with *ATG7* to receive the ubiquitin‐like molecule *ATG12*. Previous study suggested that *IRGM* may promote the nucleation and/or elongation of autophagy vesicles by interacting with one or more of *ATG10*, *ATG5*, microtubule‐associated protein 1 light chain 3 gamma (*MAP1LC3C*), and SH3 domain‐containing GRB2 like, endophilin B1 (*Sh3GLB1*).^24^ Therefore, we speculate that the correlation between *IRGM* and *ATG10* may be enhanced in AAV patients, thereby increasing individual susceptibility to AAV. However, we did not perform studies to explore how these six SNPs interact with each other and the molecular mechanisms of their interaction to increase the susceptibility to AAV, and future experimental studies will be needed.

In conclusion, there are polymorphisms in rs1864182 and rs1864183 of *ATG10* gene in Guangxi population. Although we did not report the association between *ATG10* rs1864182 and rs1864183 gene polymorphisms and AAV in our population, SNP‐SNP interactions in autophagy genes family may increase the susceptibility risk of individuals to AAV. We do believe that our results partially fill this gap, which may be useful for our understanding of the pathogenesis of AAV. Future studies are needed to investigate more autophagy genes family SNPs in a large sample to demonstrate their associations.

## CONFLICT OF INTEREST

The authors declare no conflict of interests.

## Data Availability

The data that support the findings of the current study are available from the corresponding author upon reasonable request.
